# Effect of health education intervention based on the precede-proceed model on patients with gestational diabetes mellitus: A single-center retrospective matched-cohort study

**DOI:** 10.12669/pjms.42.6.16069

**Published:** 2026-06

**Authors:** Jia Yu, Xiaoqing Zhang, Yan Chen

**Affiliations:** 1 Jia Yu, Reproductive and Fetal Care Ward, Huzhou Maternity & Child Health Care Hospital, Zhejiang Province 313000, P.R. China; 2 Xiaoqing Zhang, Department of Neonatology, Huzhou Maternity & Child Health Care Hospital, Zhejiang Province 313000, P.R. China; 3 Yan Chen, Department of Nursing, Huzhou Maternity & Child Health Care Hospital, Zhejiang Province 313000, P.R. China

**Keywords:** Gestational diabetes mellitus, Health education, PRECEDE-PROCEED Model, Pregnancy, Self-management ability

## Abstract

**Objective:**

Conventional health education may not fully address the difficulty of translating GDM-related knowledge into sustained self-management behaviors. This study aimed to evaluate whether a PRECEDE-PROCEED Model-based GDM health education program was associated with improved self-management ability and pregnancy outcomes in patients with GDM.

**Methodology:**

This single-center retrospective matched-cohort study was conducted at Huzhou Maternity & Child Health Care Hospital and included clinical data of pregnant women who underwent regular prenatal examinations in the obstetric outpatient department and were diagnosed with GDM from August 2024 to August 2025. Based on the nursing methods received, the participants were divided into an observation group (GDM health education intervention program based on the PRECEDE-PROCEED Model) and a control group (standard GDM health education intervention program).

**Results:**

A total of 150 patients were enrolled. Patients were matched at a 1:1 ratio, with 75 cases in each group. Post-intervention, fasting and two-hour postprandial blood glucose levels were lower in the observation group than in the control group (p<0.05), and two-hour postprandial blood glucose decreased after intervention in both groups. Scores of GDM-related knowledge, management behaviors, attitudes, beliefs, and social support were significantly elevated in both groups, and considerably higher in the observation group (p<0.05). The incidence of postpartum hemorrhage and hypertensive disorders of pregnancy was notably lower in the observation group (p<0.05).

**Conclusion:**

Compared with conventional health education, the GDM health education intervention program based on the PRECEDE-PROCEED Model was associated with better blood glucose levels in GDM patients, improved self-management skills, and lower incidence rates of postpartum hemorrhage and hypertensive disorders of pregnancy.

## INTRODUCTION

Incidence of gestational diabetes mellitus (GDM) is on the rise, making it one of the most prevalent metabolic diseases during pregnancy.^1^ Studies have confirmed that GDM not only increases the risk of adverse pregnancy outcomes, such as macrosomia, cesarean section, and hypertensive disorders of pregnancy, but also serves as a significant predictor for the subsequent development of Type-2 diabetes mellitus (T2DM) in women.^2^–^4^ Additionally, infants born to mothers with GDM are at an elevated risk of developing obesity and metabolic disorders later in life.^5^ Therefore, identifying effective interventions to improve glycemic control and reduce adverse pregnancy outcomes in women with GDM is essential for improving current maternal and neonatal outcomes and may also contribute to the prevention of long-term metabolic risks in mothers and offspring.^6^

Health education has been shown to be a core strategy for managing GDM. A large body of research has demonstrated that health education interventions focusing on lifestyle modifications, including appropriate dietary guidance, regular exercise, and standardized blood glucose monitoring, can effectively reduce the risk of GDM and mitigate adverse maternal and neonatal outcomes.^7^,^8^ However, traditional health education models for pregnant women often focus on knowledge dissemination, lacking systematic guidance on the motivation for behavioral changes and psychological factors, resulting in the difficulty of maintaining long-term intervention effects.^8^ The major challenge in GDM care is the difficulty of translating knowledge into sustained behavior, the limited durability of one-time education, and the interruption of management due to insufficient family and social support. Conventional education alone may be insufficient in GDM care, whereas a more systematic, sustained, family-involved, and behavior-oriented intervention may better support self-management and glycemic control.

The PRECEDE-PROCEED Model is a structured planning approach for assessing health issues for designing, implementing, and evaluating health promotion and other public health programs to meet specific needs.^9^ As a systematic and comprehensive theoretical framework for health behavior interventions, this approach emphasizes that individual behaviors are jointly influenced by predisposing, enabling, and reinforcing factors, and highlights the dynamic interactions among environmental, behavioral, and individual characteristics.^10^ Studies have shown that health promotion programs based on the PRECEDE-PROCEED Model significantly improve patients’ health knowledge.

A systematic review and meta-analysis of 26 intervention studies found that interventions using the PRECEDE-PROCEED Model are superior to conventional health education in knowledge acquisition and cognitive improvement.^11^ Furthermore, in a health intervention study involving patients with male infertility, those who received interventions based on the PRECEDE-PROCEED Model achieved significantly higher scores in lifestyle-related knowledge, health beliefs, and behaviors than the control group, with a marked improvement in adherence to healthy lifestyles.^12^ Thus, integrating the PRECEDE-PROCEED Model into health education interventions for GDM may strengthen theoretical guidance by building on existing lifestyle interventions, addressing psychological and environmental factors that influence behavioral change, overcoming the shortcomings of traditional health education and providing new research insights to enhance the effectiveness of GDM management. However, evidence supporting its use in GDM management remains limited. This study aimed to develop a GDM health education intervention program guided by the PRECEDE-PROCEED Model, explore its impacts on patients’ blood glucose control, self-management ability, and pregnancy outcomes, and provide a theoretical basis and practical reference for optimizing GDM health education models.

## METHODOLOGY

This single-center, retrospective matched-cohort study was conducted at Huzhou Maternity & Child Health Care Hospital and included clinical data on pregnant women with GDM who underwent regular prenatal checkups in the obstetric outpatient department from August 2024 to August 2025.

### Ethical approval:

The ethics committee of our hospital approved this study with the number 2024-024R, Date: May 23^rd^, 2024.

Based on the nursing interventions received, the participants were divided into an observation group, which received the PRECEDE-PROCEED Model-based health education intervention, and a control group, which received standard GDM health education. To reduce confounding from baseline differences, covariate-based rather than propensity score-based 1:1 caliper matching was performed between the two groups using the nearest-neighbor method without replacement. Matching variables included age, gestational age, gravidity, parity, pre-pregnancy body mass index (BMI), baseline glucose levels, and educational level. The caliper ranges were set as follows: age within ±2 years, pre-pregnancy BMI within ±1 kg/m^2^, and baseline glucose levels within ±0.5 mmol/L. Education level was included because it may influence the effect of health education.

### Diagnostic basis of gestational diabetes mellitus (GDM):

The diagnostic criteria for GDM in this study follow the International Association of Diabetes and Pregnancy Study Groups (IADPSG) standard.^13^ All subjects underwent a 75-g oral glucose tolerance test (OGTT) at 24–28 weeks of gestation. The detection method involved taking 75 g of glucose orally on an empty stomach in the morning. The venous plasma glucose level was measured on an empty stomach and at one hour and two hour after glucose administration. GDM can be diagnosed if the blood glucose value reaches or exceeds the corresponding threshold at any of the following points:

- Fasting blood glucose ≥ 5.1 mmol/L;
- Blood glucose ≥ 10.0 mmol/L one hour after taking glucose;
- Blood glucose ≥ 8.5 mmol/L two hours after taking sugar. Those who met any of the above indicators were included in the scope of GDM diagnosis.

### Inclusion criteria:

- GDM diagnosed based on a 75-g oral glucose tolerance test (OGTT), in accordance with established diagnostic criteria, and defined as meeting or exceeding any one of the following plasma glucose thresholds: fasting ≥5.1 mmol/L, 1-hour ≥10.0 mmol/L, or 2-hour ≥8.5 mmol/L.
- Age 18-45.
- Gestational weeks: 24-28 weeks.
- Singleton pregnancy.
- No clear history of abnormal glucose metabolism before pregnancy; GDM was found for the first time in this pregnancy.
- The patients were able to receive continuous outpatient follow-up and health education intervention during the study period, and the follow-up compliance was good.
- Have normal communication and understanding skills and can cooperate to complete the questionnaire survey and related assessment.
- The clinical data were complete, including basic demographic data, blood glucose index, self-management questionnaire, and pregnancy outcome information.

### Exclusion criteria:

- Pre-existing diabetes mellitus diagnosed before pregnancy.
- Gestational diabetes mellitus complicated by significant endocrine disorders, including hyperthyroidism or hypothyroidism, Cushing syndrome, or polycystic ovary syndrome (PCOS) with marked metabolic abnormalities.
- Severe dysfunction of major organs, including the heart, liver, kidneys, brain, or lungs, or the presence of systemic chronic diseases that could affect pregnancy outcomes or glucose metabolism.
- Presence of malignant tumors, autoimmune diseases, or active infectious diseases.
- Current or previous history of severe psychiatric disorders or cognitive impairment that could interfere with participation in health education interventions or completion of questionnaires.
- Pregnancy achieved through assisted reproductive technology.
- Long-term use during pregnancy of medications known to significantly affect glucose metabolism (e.g., glucocorticoids).
- Presence of high-risk pregnancy conditions that could substantially affect pregnancy outcomes, such as placenta previa, placental abruption, recurrent vaginal bleeding, threatened abortion, or frequent uterine contractions requiring prolonged bed rest.
- Transfer to another medical institution during the study period, loss to follow-up, or substantial missing data.

### Intervention programs:

The interventions in both groups were delivered by diabetes specialist nurses, nutrition clinic physicians or specialists, and obstetric nurses who had received standardized training before implementation. The core health education intervention started immediately after GDM diagnosis and continued for eight weeks in both groups. Nursing care during delivery hospitalization and post-discharge telephone contact were used for routine perinatal management and outcome follow-up, rather than as an additional long-term intervention phase.

### Standard GDM health education intervention:

Patients in the control group received standard GDM health education after diagnosis. The educational modules included basic knowledge of GDM, dietary control, exercise recommendations, home blood glucose monitoring, medication guidance when needed, and reminders for regular review. At the first outpatient visit after diagnosis, nutrition clinic physicians or specialists provided one-on-one guidance and distributed a general health guidance manual. During the 8-week period, patients underwent routine outpatient nutrition reviews, during which physicians provided general feedback according to BMI, blood glucose levels, and dietary records. Routine telephone follow-up was conducted once weekly, approximately 30 minutes each time, mainly to assess diet, exercise, and glucose monitoring and to remind patients to attend scheduled reviews. Family members were allowed to accompany patients during outpatient visits or participate in routine education, but family participation was not systematically organized. The control group did not receive structured WeChat-based management, daily educational content delivery, peer-support activities, or family-based reinforcement tasks.

### Health education program based on the PRECEDE-PROCEED model:

The principles of the PRECEDE-PROCEED Model-based GDM health education program are summarized in Supplementary Table-I. Patients in the observation group received one structured educational or follow-up contact each week for 8 weeks, lasting approximately 45–60 minutes per session. The intervention was organized according to predisposing, enabling, and reinforcing factors. For predisposing factors, diabetes specialist nurses provided standardized education on the causes and risks of GDM, glycemic targets, dietary principles, exercise recommendations, home glucose monitoring, and warning signs requiring medical consultation. For enabling factors, individualized diet and exercise plans were formulated according to gestational age, pre-pregnancy BMI, glucose levels, dietary habits, and individual difficulties, and were adjusted during weekly follow-up. A “Good Mom” WeChat group was used to support continuous management. Educational content was pushed once daily, including dietary reminders, exercise guidance, glucose-monitoring tips, follow-up reminders, and answers to common questions. Patients could submit questions through the WeChat group, and the healthcare team checked and responded regularly during working hours.

**Supplementary Table-I:** Intervention program.

Dimension	PRECEDE-PROCEED Model-Based GDM Health Education Intervention Program	Standard GDM Health Education Intervention Program
Intervention Basis	Guided by the PRECEDE-PROCEED Model; systematically analyzes GDM management factors from three dimensions (predisposing, enabling, reinforcing factors) to design targeted interventions.	Based on clinical guidelines and routine nursing experience; focuses on basic disease knowledge and behavior notification, lacking systematic theoretical framework support.
Core Dimension Framework	Covers three dimensions (predisposing: health education & cognition building; enabling: continuous services & resource support; reinforcing: social support & incentive feedback) to form a “cognition-behavior-support” closed loop.	Centered on “knowledge transfer + behavior guidance” with a single dimension, mainly including disease knowledge, diet, exercise, and blood glucose monitoring notification.
Specific Intervention Content	Predisposing factors: standardized education on GDM causes, risks, glycemic targets, dietary principles, exercise recommendations, home glucose monitoring, and warning signs requiring medical consultation. Enabling factors: individualized diet and exercise plans based on gestational age, pre-pregnancy BMI, glucose levels, dietary habits, and individual difficulties; plans were adjusted during weekly follow-up. A “Good Mom” WeChat group was used, with once-daily educational content push and regular responses during working hours. Reinforcing factors: spouse/family participation in education, meal planning, postprandial walking, glucose-monitoring reminders, emotional support, peer experience sharing, and positive feedback.	Standard education included basic GDM knowledge, diet, exercise, home glucose monitoring, medication guidance when needed, and review reminders. One-on-one outpatient guidance was provided after diagnosis, with distribution of a general health guidance manual. Patients received routine outpatient nutrition reviews and weekly telephone follow-up for 8 weeks. Family members were allowed to accompany patients but were not systematically involved. No structured WeChat management, daily content push, peer-support activity, or family-based reinforcement task was provided.
Implementation Approach	Structured and interactive: one educational/follow-up contact per week for 8 weeks, 45–60 minutes each; daily WeChat content delivery; regular online response during working hours; outpatient review; peer sharing; and family-supported management.	Routine and less interactive: one-on-one outpatient education at diagnosis, general written materials, routine outpatient nutrition reviews, and weekly telephone follow-up for 8 weeks, approximately 30 minutes each time.
Engagement and Completion Assessment	Engagement was assessed by weekly follow-up completion, attendance at educational contacts, submission of glucose records, WeChat interaction or reading feedback, and completion of pre- and post-intervention questionnaires.	Completion was assessed by weekly telephone follow-up completion, outpatient review attendance, and completion of pre- and post-intervention questionnaires.
Personalization Level	Highly personalized: formulates diet/exercise plans based on gestational weeks, weight, blood glucose levels, and eating habits; dynamically adjusts per recheck results; provides solutions for individual difficulties.	Standardized: adopts unified health guidance content, rarely considering individual differences (e.g., underlying diseases, lifestyle, family environment).
Social Support Integration	Emphasizes tripartite support (family, peers, medical staff): invites family participation in education, establishes peer support groups, real-time online response from medical staff to form a multi-dimensional support network.	Dominated by medical guidance: rarely involves family/peer participation, lacking social support dimensions.
Intervention Objectives	Multi-dimensionally improves self-management ability through “cognition-behavior-support”; consolidates healthy behaviors long-term; reduces pregnancy complication risks.	Mainly targets short-term mastery of basic management skills and blood glucose control; lacks focus on sustained behavior maintenance.

For reinforcing factors, spouses or family members were invited to participate in health education and were encouraged to assist with meal planning, accompany patients during postprandial walking, remind patients to monitor blood glucose, and provide emotional support. Peer experience sharing and positive feedback were also used to reinforce adherence. Participant engagement and completion were assessed according to weekly follow-up completion, attendance at educational contacts, submission of glucose monitoring records, WeChat interaction or reading feedback, and completion of the pre- and post-intervention questionnaires. Completion of the intervention was defined as completing the 8-week health education program and both pre- and post-intervention assessments.

### Data collection:

The basic patient characteristics, baseline and post-intervention blood glucose values, GDM self-management behavior score, and maternal and infant outcome indicators were collected from the electronic medical record system. The primary outcomes were glycemic control and improvement in self-management ability. The secondary outcomes were selected maternal complications, including postpartum hemorrhage and hypertensive disorders of pregnancy. Macrosomia and fetal growth restriction were prespecified as exploratory neonatal outcomes because of their low event rates. The operational definitions of adverse pregnancy outcomes were as follows. Postpartum hemorrhage was defined as blood loss ≥500 mL after vaginal delivery or ≥1000 mL after cesarean section within 24 hours after delivery, or the presence of signs and symptoms of hypovolemia.. Hypertensive disorders of pregnancy included chronic hypertension in pregnancy, hypertensive disorders of pregnancy, preeclampsia-eclampsia, and preeclampsia superimposed on chronic hypertension. Infection included chorioamnionitis, genital Chlamydia trachomatis infection, vaginal group B streptococcal infection, vulvovaginal candidiasis, and Mycoplasma infection during pregnancy. Hypokalemia was defined as a serum potassium level of less than 3.5 mmol/L. Macrosomia was defined as a birth weight greater than 4000 g. Fetal growth restriction was defined as an estimated fetal weight or abdominal circumference below the 10th percentile for gestational age. The GDM self-management questionnaire was used to assess the self-management abilities of GDM patients. The questionnaire was compiled by Qimengjun and consisted of 26 items across four dimensions: GDM-related knowledge, GDM management methods and behavior, GDM attitude and belief, and social support. The questionnaire items are scored on a Likert scale: 1 = very disagree, 2 = disagree, 3 = not necessarily, 4 = agree, and 5 = very agree.

### Statistical analysis:

SPSS statistical software (version 26.0; IBM Corp, Armonk, NY, USA) for Windows was used. The matching procedure was implemented in SPSS version 26.0 using covariate-based nearest-neighbor caliper matching without replacement. Only successfully matched pairs were included in the final comparative analysis, whereas unmatched cases were excluded. Post-matching covariate balance was assessed using standardized mean differences (SMDs), which are reported in Table-I. The count data were expressed as n (%). The differences between groups were tested by the chi-square test or Fisher’s exact test, such as education level and adverse pregnancy outcomes. Visual (histogram and probability map) and analytical (Kolmogorov-Smirnov/Shapiro-Wilk test) methods were used to evaluate whether continuous variables obey a normal distribution. The measurement data, which were normally distributed, were expressed as mean ± standard deviation (SD). The difference between the two groups was tested using an independent-samples t-test, and a paired t-test was used to compare within-group differences. Data with a non-normal distribution were reported as median and interquartile interval. The Mann-Whitney U test was used to compare groups, and the Wilcoxon signed-rank test was used to compare within-group differences. Statistical significance was set as *P*<0.05.

**Table-I:** Comparison of basic characteristics between the two groups.

Characteristics	Observation group (n=75)	Control group (n=75)	t/Z/χ^2^	P	SMD
Age (years)	30 (27-33)	32 (28-34)	-1.365	0.172	0.209
Gestational age	26 (25-27)	26 (25-27)	-0.877	0.380	0.148
Number of pregnancies	2 (1-2)	2 (1-3)	-1.096	0.273	0.127
Parity	1 (0-1)	1 (0-1)	-0.864	0.388	0.104
Baseline fasting blood glucose (mmol/l)	4.8 (4.48-5.17)	4.91 (4.56-5.26)	-1.120	0.263	0.221
Baseline 1h postprandial blood glucose (mmol/l)	10.2 (9.37-11.08)	10.13 (8.99-10.85)	-0.584	0.559	0.026
Baseline 2h postprandial blood glucose (mmol/l)	8.63±1.75	8.56±1.53	0.257	0.797	0.042
Pre pregnancy BMI (kg/m ^2^)	23.2 (21.2-25)	23.6 (21.6-25.1)	-0.624	0.533	0.136
Degree of education			0.107	0.743	0.053
Junior high school and below	37 (49.3)	33 (44.0)			
High school and above	38 (50.7)	42 (56.0)			

***Note:*** SMD, standardized mean difference. SMD values were calculated after matching to assess covariate balance between the two groups.

## RESULTS

A total of 713 patients were screened according to the inclusion and exclusion criteria, of whom 86 were excluded. Before matching, 292 patients were assigned to the observation group and 335 patients to the control group. After covariate-based 1:1 nearest-neighbor caliper matching without replacement, 75 patients in the observation group were matched with 75 patients in the control group and included in the final analysis. Unmatched cases were excluded from the final comparative analysis. The final matched cohort included 150 patients with GDM aged 20–42 years, with a median age of 30.5 years [interquartile range, 28–34 years]. The post-matching SMD values for baseline variables are presented in Table-I. All SMD values were below 0.25, suggesting no marked baseline imbalance between the two groups, although minor residual imbalance remained for age and baseline fasting blood glucose.

Before intervention, there was no significant difference in fasting blood glucose and two hours postprandial blood glucose levels between the two groups (*P*>0.05). After the intervention, fasting blood glucose and two hours postprandial blood glucose levels were significantly lower in the observation group than in the control group (P<0.05). The two hours postprandial blood glucose level decreased after intervention in both groups, with a greater reduction observed in the observation group (Table-II).

**Table-II:** Comparison of blood glucose levels between the two groups before and after intervention.

Variables	Observation group (n=75)	Control group (n=75)	t/Z	P
***Before intervention***				
Fasting blood glucose (mmol/l)	4.8 (4.48-5.17)	4.91 (4.56-5.26)	-1.120	0.263
2h postprandial blood glucose (mmol/l)	8.63±1.75	8.56±1.53	0.257	0.797
***After intervention***				
Fasting blood glucose (mmol/l)	4.87 (4.68-5.23)*	5.24 (5.02-5.58)*	-4.928	<0.001
2h postprandial blood glucose (mmol/l)	6.52 (5.83-7.99)*	7.78 (7.03-8.6)*	-4.227	<0.001

***Note:*** compared with before intervention in the same group,

* P<0.05.

As shown in Table-III, there were no significant differences in pre-intervention GDM-related knowledge, GDM management methods and behaviors, attitudes and beliefs, and social support scores between the two groups (*P*>0.05). After the intervention, GDM-related knowledge, GDM management methods and behaviors, attitudes and beliefs, and social support scores of all patients significantly improved, and were markedly higher in the observation group compared to the control group (*P*<0.05).

**Table-III:** Comparison of GDM self-management scores between the two groups before and after intervention.

Variables	Observation group (n=75)	Control group (n=75)	Z	P
Before intervention				
GDM related knowledge	28 (26-34)	27 (25-32)	-1.788	0.074
GDM management methods and behaviors	26 (25-29)	26 (23-30)	-1.035	0.301
Attitude and belief	15 (13-16)	15 (13-17)	-1.339	0.180
Social support	15 (13-16)	14 (12-15)	-1.941	0.052
After intervention				
GDM related knowledge	33 (30-38)*	29 (27-34)*	-4.558	<0.001
GDM management methods and behaviors	30 (29-34)*	28 (26-32)*	-3.988	<0.001
Attitude and belief	19 (17-20)*	17 (15-19)*	-4.039	<0.001
Social support	18 (17-19)*	16 (15-17)*	-5.551	<0.001

***Note:*** compared with before intervention in the same group,

* P<0.05.

The reported adverse pregnancy outcomes included 70 cases of cesarean section (46.7%), 60 cases of postpartum hemorrhage (40.0%), and 41 cases of infection (27.3%). The incidence of postpartum hemorrhage and hypertensive disorders of pregnancy in the observation group was significantly lower than that in the control group (*P*<0.05). For the exploratory neonatal outcomes, four cases of macrosomia (2.7%) and six cases of fetal growth restriction (4.0%) were recorded, with no significant between-group differences (*P*>0.05; Table-IV).

**Table-IV:** Comparison of adverse pregnancy outcomes between the two groups.

Adverse pregnancy	Observation group (n=75)	Control group (n=75)	χ^2^	P
Cesarean section	34 (45.3)	36 (48.0)	0.107	0.743*
Postpartum hemorrhage	21 (28.0)	39 (52.0)	9.000	0.003*
Hypertensive disorders of pregnancy	1 (1.3)	8 (10.7)	―	0.034**
Infection	17 (22.7)	24 (32.0)	1.645	0.200*
Hypokalemia	7 (9.3)	6 (8.0)	0.084	0.772*
Premature rupture of membranes	3 (4.0)	2 (2.7)	―	1.000**
Macrosomia	1 (1.3)	3 (4.0)	―	0.620**
Fetal growth restriction	3 (4.0)	3 (4.0)	―	1.000**

* Pearson’s Chi-square test;

** Fisher’s Exact Test.

## DISCUSSION

Given the challenges in sustaining behavioral change, the limited durability of one-off education, and interruptions caused by inadequate family and social support, GDM care requires a more systematic and continuous approach. The results of this study showed that compared with standard health education, the PRECEDE-PROCEED Model-based intervention was associated with improved patients’ blood glucose control. Both FPG and 2hPG levels in the observation group were significantly lower than those in the control group. Additionally, this intervention model demonstrated greater benefits in enhancing self-management skills among patients with GDM, as evidenced by comprehensive improvements across multiple dimensions (disease-related knowledge, management behaviors, attitudes and beliefs, and social support). Compared with standard health education, the PRECEDE-PROCEED Model-based intervention was also associated with a lower incidence of selected secondary maternal outcomes, including postpartum hemorrhage and hypertensive disorders of pregnancy, whereas no significant between-group difference was observed in the exploratory neonatal outcomes. These findings suggested that the PRECEDE-PROCEED Model-based health education intervention has potential clinical value in optimizing self-management behaviors and improving certain maternal pregnancy outcomes among patients with GDM, which may contribute to postpartum and long-term metabolic health. These findings align with the previous studies on GDM health education interventions.^14^,^15^

Studies have pointed out that blood glucose management in GDM patients is critical for maternal and infant health outcomes, noting that GDM is associated with outcomes such as macrosomia and cesarean section.^16^–^19^ In this study, post-intervention FPG was lower in the observation group than in the control group, although a within-group reduction in FPG from baseline was not observed. By contrast, 2hPG decreased after intervention, with a greater reduction in the observation group. These findings suggest that the PRECEDE-PROCEED Model-based intervention may be particularly beneficial for improving postprandial glycemic control.. Studies on the PRECEDE-PROCEED Model have shown that through systematic behavior change strategies, this framework can significantly improve patients’ adherence to blood glucose monitoring, thereby enhancing blood glucose control.^20^ In addition, research has emphasized the impact of nutrient intake on blood glucose control, noting that a higher carbohydrate-to-energy ratio is negatively correlated with poor blood glucose control and suggesting that dietary structure adjustment is key to blood glucose management.^21^ Therefore, the systematic intervention based on the PRECEDE-PROCEED Model in this study not only strengthened patients’ theoretical cognition but also achieved effective blood glucose control through behavior changes, such as dietary adjustment and regular exercise.

This study also showed that patients in the intervention group had higher self-management scores across the dimensions of GDM-related knowledge, management behaviors, attitudes and beliefs, and social support. These improvements may provide a behavioral explanation for the observed differences in glycemic control. Greater disease-related knowledge may help patients recognize the risks of high-glycemic dietary patterns and understand the importance of regular glucose monitoring. Improved management behaviors may translate into more consistent dietary control, postprandial physical activity, and home glucose monitoring. In addition, more positive attitudes and stronger health beliefs may reduce non-adherent perceptions, such as the belief that occasional relaxation of dietary or monitoring requirements has little consequence, thereby supporting sustained self-management.^8^,^18^,^22^,^23^ Social support is also clinically relevant in GDM care, because family members often influence meal preparation, daily activity, emotional state, and adherence to follow-up. Stronger family and social support may therefore reduce family-level barriers to dietary control and postprandial activity, particularly when family members participate in meal planning, share appropriate dietary patterns, and encourage regular glucose monitoring. Through these cognitive, behavioral, psychological, and social pathways, the PRECEDE-PROCEED Model-based intervention may contribute to more stable glycemic control and may partly explain the lower incidence of selected maternal complications observed in the intervention group. This interpretation is consistent with previous studies showing that theory-based educational interventions can enhance self-efficacy, health beliefs, and self-management behaviors.^8^,^24^–^26^

In addition, this study found that the incidence of postpartum hemorrhage and hypertensive disorders of pregnancy in the intervention group was significantly lower than in the control group. Although existing studies have pointed out that poor blood glucose control is associated with outcomes such as hypertensive disorders of pregnancy and postpartum hemorrhage,^17^,^27^,^28^ there is still insufficient evidence on the impact of interventions on these outcomes. However, several studies suggested that systematic health education interventions (such as the PRECEDE-PROCEED Model) may reduce the risk of pregnancy complications by reducing blood glucose fluctuations and alleviating vascular endothelial damage.^14^,^20^,^29^

No significant between-group differences were observed in the exploratory neonatal outcomes, including macrosomia and fetal growth restriction. These findings should be interpreted cautiously because the number of neonatal events was small and the study was not primarily powered to detect differences in these outcomes. Although maternal glycemic control is closely related to fetal growth and neonatal weight, neonatal outcomes are also influenced by multiple factors, including baseline maternal metabolic status, genetic background, placental function, and obstetric management. Therefore, the absence of statistically significant differences in macrosomia or fetal growth restriction should be regarded as preliminary rather than definitive evidence. Larger prospective studies with adequate power are needed to determine whether model-based health education can produce measurable benefits in neonatal outcomes.

### Strengths of the study:

The major strength of this study lies in its application of the PRECEDE-PROCEED model to GDM health education and its simultaneous evaluation of behavioral indicators and clinical outcomes in a matched cohort.

### Limitations:

However, several limitations should be acknowledged. First, this was a single-center retrospective matched-cohort study rather than a randomized trial. Although 1:1 matching was used to reduce baseline differences between the two groups, the observational design could not fully eliminate selection bias, information bias, or residual confounding. Information bias may have arisen from retrospective data extraction from electronic medical records, incomplete or inconsistent follow-up documentation, and the use of self-reported questionnaires to assess self-management ability. Therefore, the findings should be interpreted as associations rather than definitive evidence of a causal intervention effect. In addition, the observed between-group differences may reflect not only the theoretical structure of the PRECEDE-PROCEED model, but also the greater continuity of contact, individualized feedback, and organized family support embedded in the model-based intervention. This should be considered when interpreting the specific contribution of the theoretical framework itself. Second, the sample size was relatively small, particularly for low-frequency neonatal outcomes such as macrosomia and fetal growth restriction; therefore, the study may have been underpowered to detect between-group differences in these exploratory outcomes. Third, the intervention and follow-up period was relatively short, covering mainly the second and third trimesters and the perinatal period. As a result, this study could not evaluate postpartum glucose status, long-term maternal metabolic risk, or long-term offspring outcomes. Finally, because the study was conducted in a single center with patients from a relatively concentrated regional and healthcare background, the generalizability of the findings should be further verified in larger multicenter prospective studies.

## CONCLUSION

Compared with conventional health education, the PRECEDE-PROCEED Model-based health education intervention was associated with better blood glucose control in patients with GDM, improved patients’ self-management capacity in terms of disease cognition, management behaviors, attitudes and beliefs, as well as social support, and a lower incidence of selected maternal complications, including postpartum hemorrhage and hypertensive disorders of pregnancy. However, these findings should be further verified in multi-center, prospective, randomized studies.

### Authors’ contributions:

**JY and YC:** Literature search, study design and manuscript writing.

**JY, YC and XZ:** Data collection, data analysis and interpretation, Critical Review.

**JY and YC:** Manuscript revision and validation and is responsible for the integrity of the study.

All authors have read and approved the final manuscript.
